# Flipping Tickborne Illnesses with Infographics

**DOI:** 10.21980/J83H12

**Published:** 2023-04-30

**Authors:** Daniel Johnson, Annahieta Kalantari

**Affiliations:** * Penn State Health Milton S Hershey Medical Center, Department of Emergency Medicine, Hershey, PA

## Abstract

**Audience:**

This interactive module is designed for implementation within an Emergency Medicine Residency program. The target audience is post-year-graduate one to post-year-graduate four residents, medical students, physician assistant postgraduate trainees, physician assistant students, and physician assistants.

**Introduction:**

A knowledge of tickborne illness represents a critical component of infectious disease education for Emergency Medicine residents. Ticks that harbor these organisms are highly endemic to the continental United States and zoonotic infections are a critical differential diagnosis in the evaluation of patients in the Emergency Department.^1^ There is significant morbidity and mortality associated with tickborne diseases, and many of the signs and symptoms can mimic other common presentations. While these illnesses can present a diagnostic challenge and coinfection does occur, treatment is generally straightforward and readily available.^2^ An understanding of vectors and rates of transmission in a geographic area can foster a high clinical suspicion and ensure that effective treatment is administered.^3^

**Educational Objectives:**

After participation in this module, learners will be able to 1) list the causative agents for Lyme Disease, Babesiosis, Tularemia, Ehrlichiosis, Anaplasmosis, Tick Paralysis, Rocky Mountain Spotted Fever, and Powassan Virus, 2) identify different clinical features to distinguish the different presentations of tickborne illnesses, and 3) provide the appropriate treatments for each illness.

**Educational Methods:**

This module utilized the flipped classroom model of education for independent learning, along with small group discussion as the in-class active learning strategy. Learners independently completed pre-assigned readings and questions based on the readings. In didactics sessions, learners created an infographic of each of the tickborne illnesses. Each infographic was shared with the entire group in the final 30 minutes of the didactic session.

**Research Methods:**

Each learner completed a pre-test prior to receiving the educational preparatory materials. At the end of the session, participants completed a post-test, a Likert scale survey to evaluate the program, and a free text box to provide qualitative feedback on the session. Efficacy of the education content was determined by post-test scores.

**Results:**

Unfortunately, the pre-test file was corrupted by a virus and inaccessible, resulting in no comparison data. A post-course test of 4 questions and a Likert scale evaluation was completed by 22 participants. 72.7% of the participants felt the session increased his/her knowledge on the topic, and 59% enjoyed the format of the session. Fifty-percent of the participants missed zero post-course test questions, 27% missed one question, and 22% missed two or more questions. Comments for improvement suggested a better explanation on the use of software to create the infographics.

**Discussion:**

The post-course test and evaluation suggest the session achieved positive Kirkpatrick levels I and II of evaluation, was effective, and the objectives were met. Based on comments for improvement, information on the infographic software should be provided ahead of the session. This session has become a regular part of our 18-month residency didactic curriculum.

**Topics:**

Infectious disease, tickborne illness, zoonosis, flipped classroom, infographic, cognitivism.

## USER GUIDE

**Table t1-jetem-8-2-sg1:** 

**List of Resources:**	
Abstract	1
User Guide	3
Small Groups Learning Materials	6
Appendix A: Individual Learner Pre-Didactic Worksheet	6
Appendix B: Small Group Application Exercise	7
Appendix C: Individual Learner Pre-Didactic Worksheet Answers	8
Appendix D: Flipping Tickborne Illnesses with Infographic Debriefing Guide	11

[Table t1-jetem-8-2-sg1] **Learner Audience:**

Medical Students, Interns, Junior Residents, Senior Residents, Physician Assistant Students

**Time Required for Implementation:**

90 minutes

**Recommended Number of Learners per Instructor**:

This is an asynchronous module, not requiring moderation in real-time.

**Topics:**

Infectious disease, tick-borne illness, zoonosis, flipped classroom, infographic, cognitivism.

**Objectives:**

After participation in this module, learners will be able to:

1. List the causative agents for Lyme Disease, Babesiosis, Tularemia, Ehrlichiosis, Anaplasmosis, Tick Paralysis, Rocky Mountain Spotted Fever, and Powassan Virus.
2. Identify different clinical features to distinguish the different presentations of tickborne illnesses.
3. Provide the appropriate treatments for each illness.

### Linked objectives and methods

Traditionally, didactics have been delivered via an hour-long lecture format. There is little data to support this as the standard form of information delivery.^4^ In the lecture format, learners are in a passive state, receiving information.^5^ For this session, we desired to have learners engaged in active learning and discussion. In order to achieve this, we utilized a flipped classroom with a small groups format. Two weeks prior to the scheduled didactic day, learners received preselected readings and a list of open-ended questions to answer based on the assigned readings. On the didactic day, learners were divided into eight different groups based on level of training with the goal of evenly distributed experience across each group.

Once divided into groups, each group was assigned one of the eight types of tickborne illnesses included in the pre-readings. Each group then created an infographic on that assigned illness. The infographics included salient information such as risk factors, vectors, presenting signs and symptoms, diagnostic testing, treatment, and complications (objectives 1, 2 and 3). The format of an infographic allowed for integration of the required information into an organized, creative, and visually stimulating representation of each illness. Infographics have been successfully used in education not only when used as the main teaching tool by educators, but also when learners have created them as part of their learning.^6^ Groups were given 45 minutes to complete the infographic. In the remaining time, the groups presented their infographics to demonstrate their learning and reinforce teaching points to the rest of the groups participating in the session.

Cognitivism is the main educational framework that guides this didactic session. In cognitivism, learners are asked to receive, process, interpret, and assign information.^7^ In this session, learners receive information in the pre-didactic readings. The processing, interpretation, and assignment of information occurs in the creation of the infographic. This learning is further reinforced when each group presents their infographic (objectives 1, 2 and 3).

### Recommended pre-reading for instructor

- Cabrera, D. Tick season is now upon us! EMBlog Mayo Clinic. June, 2015. Available at: http://emblog.mayo.edu/2015/06/15/tick-season-is-now-upon-us/
- Biggs HM, Behravesh CB, Bradley KK, et al. Diagnosis and Management of Tickborne Rickettsial Diseases: Rocky Mountain Spotted Fever and Other Spotted Fever Group Rickettsioses, Ehrlichioses, and Anaplasmosis — United States. *MMWR Recomm Rep* 2016;65(No. RR-2):1–44. DOI:http://dx.doi.org/10.15585/mmwr.rr6502a1
- Eisen R, Paddock C, Ebel GD, Pritt BS. Emerging Tickborne Diseases. (Video.) YouTube. July, 2019. Centers for Disease Control and Prevention. Available at: https://www.cdc.gov/grandrounds/pp/2017/20170321-tickborne-diseases.html
- Farkas, J. Tick-borne illnesses. EMCrit Project. 2022. Available at: https://emcrit.org/ibcc/tick/
- Pace EJ, O’Reilly M. Tickborne diseases: Diagnosis and management. *Am Fam Physician.* 2020;101(9):530–540. Available at: https://www.aafp.org/pubs/afp/issues/2020/0501/p530.html
- Tickborne Diseases of the United States: A Reference Manual for Health Care Providers. 2022. Centers for Disease Control and Prevention. Available at: https://www.cdc.gov/ticks/tickbornediseases/TickborneDiseases-P.pdf

### Learner responsible content (LRC)

- Cabrera, D. Tick season is now upon us! EMBlog Mayo Clinic. June, 2015. Available at: http://emblog.mayo.edu/2015/06/15/tick-season-isnow-upon-us/
- Biggs HM, Behravesh CB, Bradley KK, et al. Diagnosis and Management of Tickborne Rickettsial Diseases: Rocky Mountain Spotted Fever and Other Spotted Fever Group Rickettsioses, Ehrlichioses, and Anaplasmosis — United States. *MMWR Recomm Rep* 2016;65(No. RR-2):1–44. DOI:http://dx.doi.org/10.15585/mmwr.rr6502a1
- Eisen R, Paddock C, Ebel GD, Pritt BS. Emerging Tickborne Diseases. (Video.) YouTube. July, 2019. Centers for Disease Control and Prevention. Available at: https://www.cdc.gov/grandrounds/pp/2017/20170321-tickborne-diseases.html
- Farkas, J. Tick-borne illnesses. EMCrit Project. 2022. Available at: https://emcrit.org/ibcc/tick/
- Pace EJ, O’Reilly M. Tickborne diseases: Diagnosis and management. *Am Fam Physician.* 2020;101(9):530–540. Available at: https://www.aafp.org/pubs/afp/issues/2020/0501/p530.html
- Tickborne Diseases of the United States: A Reference Manual for Health Care Providers. 2022. Centers for Disease Control and Prevention. Available at: https://www.cdc.gov/ticks/tickbornediseases/TickborneDiseases-P.pdf

### Results and tips for successful implementation

A pre-test and post-course test and evaluation measured this didactic at Kirkpatrick levels I and II.^8^ The pre and post-test consisted of the same 4 multiple choice questions. Four questions were included to keep the overall number of survey questions low to aid response rate. The post-course evaluation included a Likert scale in addition to an open text box to provide qualitative feedback comments.

A total number of 22 learners participated in the didactic session. Unfortunately, the pre-test file was corrupted by a virus and inaccessible, resulting in no comparison data. The post-course test and evaluation was completed by all 22 participants. Seventy-three perecent of the participants felt the session increased his/her knowledge on the topic, and 59% enjoyed the format of the session. Fifty percent of the participants missed zero post course test questions, 27% missed one question, and 22% missed two or more questions.

Open text box suggestions provided varied comments. Most learners commented that they enjoyed the didactic session. One comment suggested receiving the prereading materials earlier than two weeks but did not offer an exact time. Most critical comments suggested a better explanation of how to use the infographic software. One group had technical challenges using the software and felt they wasted time figuring out the software when they could have been working on the assignment. In the future, we plan to send a link of a video on how to use the software prior to the didactic session so learners have time to familiarize themselves with the software. Familiarity with the program may also ensure that the exercise can be completed in the allotted 45 minutes. All the information required to create the infographics is available in the pre-session questions; thus, time spent on searching for salient information is greatly decreased if particpants prepared for the session.

Also, given the availability of the online platform, the notion of creating a portion of the infographic asynchronously could allow more time for in-class discussion; however, working in small groups outside of allotted conference time may be logistically challenging. Providing an example infographic could be another means to portray expectations and increase the efficiency of the process.

Lastly, each program is able to cater this session with regard to time and group numbers to their needs. Although we utilized 45 minute sessions and eight small groups, other programs may wish to allot additional time for the creation of the infographic or consolidate groups to a lower number of groups.

### Associated content/sections of didactics

Appendix A: Individual Learner Pre-Didactic Worksheet
Appendix B: Small Group Application Exercise
Appendix C: Individual Learner Pre-Didactic Worksheet Answers
Appendix D: Flipping Tickborne Illnesses with Infographic Debriefing Guide

### Pearls

Learners receive the answers to the worksheet they were required to complete prior to the session. No handouts were given.

## SMALL GROUPS LEARNING MATERIALS

### Appendix A Individual Learner Pre-Didactic Worksheet

1. What are the three major ticks of concern in the United States that cause human disease?
2. In what regions of the United States are the three major ticks endemic?
3. What disease is each of the three major ticks responsible for transmitting to humans?
4. What are three main categories of prevention strategies?
5. In terms of the treatment of rickettsial illnesses, what age groups should not be treated with doxycycline?
6. What is Powassan virus?
7. What are two other emerging tickborne viruses?
8. What is the primary form of diagnostic testing used for most tickborne nonviral diseases?
9. How many weeks after infection does serology become an effective form of testing for nonviral tickborne illnesses?
10. What is the primary form of diagnostic testing for Babesiosis?
11. Discuss the use of nucleic acid amplification tests in the detection of *Anaplasma*, *Ehrlichia* and *Babesia* species?
12. What is the best way to remove a tick?
13. What repellants work best to prevent tick exposures?
14. How long does it take for a tick to transmit Lyme disease?
15. In what setting are prophylactic antibiotics recommended?
16. What are the four clinical patterns associated with tickborne illnesses and the illnesses associated with them?
17. What are the treatments of choice for each of the above illnesses?

### Appendix B Small Group Application Exercise (sGAE)

Create an infographic of your assigned tickborne illness. Please include the geographic distribution, vector, clinical presentation, and treatment in your infographic. You may use any infographic software available. If you are unfamiliar with any existing software, Piktochart is available at https://piktochart.com/ and is free of charge. Be prepared to present your infographic to the rest of the group.

### Appendix C Individual Learner Pre-Didactic Worksheet Answers

1. What are the three major ticks of concern in the United States (US) that cause human disease?^9^
  - *Ixodes scapularis* (Deer tick, Blacklegged tick)
  - *Amblyomma americanum* (Lone star tick)
  - *Dermacentor variabilis* (American dog tick)
2. In what regions of the United States are the three major ticks endemic?^9^
  - *Ixodes scapularis* – woodlands, Eastern US.
  - *Amblyomma americanum* – predominantly southeastern US
  - Dermacentor variabilis – broadly distributed on the east and west coasts of the US.
3. What disease is each of the three major ticks responsible for transmitting to humans?^9^
  - *Ixodes scapularis*
    - Anaplasmosis
    - Babesiosis
    - *Borrelia miyamtoi* disease
    - Ehrlichiosis
    - Lyme disease
    - Powassan encephalitis
  - *b. Amblyomma americanum*
    - Ehrlichiosis
    - Tularemia
    - Heartland virus disease
  - *c. Dermacentor variabilis*
    - Rocky Mountain Spotted Fever (RMSP)
    - Tularemia
4. What are three main categories of prevention strategies?^9^–^12^
  - Personal protection: long-sleeved and/or permethrin treated clothing, insect repellant containing N, N-Diethyl-meta-toluamide (DEET), and personal surveillance for ticks after being outdoors.
  - Environmental modification
  - Tick suppression
5. In terms of the treatment of rickettsial illnesses, what age groups should not be treated with doxycycline?
  - There is no recommended age limit for treatment with doxycycline. Todd et all demonstrated no association between the use of doxycycline and tooth staining.^13^
6. What is Powassan virus?^9^,^14^
  - Powassan virus is a flavivirus that was first discovered in 1958 and is considered an emerging tickborne disease in the US. It has a rodent reservoir and carries a 25% case fatality rate if encephalitis develops.
7. What are two other emerging tickborne viruses?^9^,^14^
  - Heartland virus: a phlebovirus transmitted by the lone star tick in the southern and southeastern US.
  - Bourbon virus: a thogotovirus whose sole case was responsible for a death in Kansas.
8. What is the primary form of diagnostic testing used for most tickborne nonviral diseases?^10^
  - Serology testing
9. How many weeks after infection does serology become an effective form of testing for nonviral tickborne illnesses?^9^,^10^
  - Approximately 1–2 weeks
10. What is the primary form of diagnostic testing for Babesiosis?^9^,^11^
  - Blood smear
11. Discuss the use of nucleic acid amplification tests in the detection of *Anaplasma*, *Ehrlichia*, and *Babesia* species.^9^
  - These tests can detect disease if the organisms are present in high amounts. Results will coincide with clinical presentation of symptoms.
12. What is the best way to remove a tick?^9^,^10^,^15^
  - Grasp the tick as close to the skin as possible with the use of fine tipped tweezers and pull with constant and steady upward pressure.
13. What repellants work best to prevent tick exposures?^11^,^12^
  - 20–30% DEET
  - Permethrin treated clothing
14. How long does it take for a tick to transmit Lyme disease?^9^
  - 36 hours
15. In what setting are prophylactic antibiotics recommended?^9^,^11^,^12^
  - Prophylaxis for Lyme disease is recommended within 72 hours if the tick has been attached for greater than 36 hours. A single dose of 200 mg of doxycycline is the recommended treatment. Prophylaxis is not recommended for children under the age of 8 and pregnant women.
16. What are the four clinical patterns associated with tickborne illnesses and the illnesses associated with them?^10^–^12^
  - Localized rash with or without fever.^11^
    - Lyme disease: can present with erythema migrans (EM) rash and associated flu-like symptoms which generally occur one to two weeks after tick bite.
    - Ulcero-glandular tularemia : tularemia can also present with rash, generally without associated flu-like syndrome. The rash is erythematous but differentiated from EM with presence of necrosis or eschar. The rash typically occurs three to five days after tick bite. Treatment may gentamycin if severe symptoms. Treatment is 14 days of doxy 100 mg po bid.
  - Febrile illness without rash
    - Anaplasmosis and Erhlichiosis^10^,^11^
      1. Can present with fever and flu-like illness without a rash. Generally occurs one to two weeks after exposure and more severe disease is seen in older and immunocompromised patient. Patient can have protracted fever for weeks to months without any other obvious source. Lab testing reveals leukopenia, lymphopenia, thrombocytopenia and a mild transaminitis. Treatment is with doxycycline.
    - Babesiosis^10^
      1. Patients generally have flu-like symptoms with high fever that occurs one to nine weeks after exposure. Labs will generally show hemolytic anemia, thrombocytopenia, renal failure, and a mild transaminitis. The hallmark of diagnosis is the Maltese cross seen on peripheral blood smear. Treatment includes atovaquone and azithromycin. May require multidisciplinary care with hematology and infectious disease.
  - Fever with a generalized rash
    - Disseminated Lyme^11^
    - RMSF^10^
      1. Typically presents with high fever, flu-like illness and gastrointestinal symptoms that occur 2–14 days after exposure. Rash is characteristic with pruritic macules and begins on the wrists and ankles and spreads to the trunk. Rash may be absent in up to 10% of cases. A petechial rash may develop through the course of illness and is indicative of severe disease. Labs are not generally helpful. Treatment is with doxycycline and this disease can be fatal without treatment.
  - Acute weakness
    - Disseminated Lyme^10^
    - Tick paralysis: caused by the dog tick most commonly in the US. Results in neurologic deficits including paresthesia (generally without objective sensory abnormalities) and ascending paralysis. Treatment is removal of the tick. This is usually curative but symptoms can progress after removal and can include respiratory musculature therefore warranting a period of observation.
17. What are the treatments of choice for each of the above illnesses?^10^,^12^
  - All are treated with doxycycline with the important exception of babesiosis (atovaquone and azithromycin) and Powassan virus (supportive care).

### Appendix D Flipping Tickborne Illnesses with Infographic Debriefing Guide

**Table t2-jetem-8-2-sg1:**

Disease: Lyme Disease (*Borrelia burgdorferi*)	
Vector	*I. scapularis*
Endemic Area	Northeastern and Midwest US
Incubation Period	3–30 Days
Signs and Symptoms	**Localized**: Erythema migrans **Disseminated**: multiple skin lesions, arthritis (migratory), cardiac (conduction abnormalities, pericarditis, myocarditis), neurologic (Bell’s palsy, meningitis)
Lab Findings	Elevated inflammatory markers Transaminitis Cerebrospinal Fluid (CSF): lymphocytic pleocytosis
Diagnosis	Antibody testing (IgM or IgG)
Treatment/Prophylaxis	**Preferred**: doxycycline **Alternatives**: cefuroxime, amoxicillin **Prophylaxis:** (age ≥ 8, endemic area, attachment ≥ 36 hours, can be initiated within 72 hours of tick removal): doxycycline

**Table t3-jetem-8-2-sg1:**

Disease: Babesiosis (*B. microti*)	
Vector	*I. scapularis*
Endemic Area	Northeastern and Midwest US
Incubation Period	1–9 weeks
Signs and Symptoms	Fevers, chills, fatigue, myalgia/arthralgia, nausea, splenomegaly, hepatomegaly
Lab Findings	Hemolytic anemia Thrombocytopenia Elevated BUN, Creatinine Transaminitis
Diagnosis	Visually on peripheral smear PCR (Polymerase Chain Reaction) Antibody testing
Treatment/Prophylaxis	Atovaquone + Azithromycin (or) Clindamycin + Quinine Quinine + Clindamycin preferred for severe disease (organ dysfunction, > 10% parasite load)

**Table t4-jetem-8-2-sg1:**

Disease: Tularemia (*F. tularensis*)	
Vector	*D. varabilis* (dog tick), *D. andersoni* (wood tick), *A. americanum* (lone star tick)
Endemic Area	Widespread
Incubation Period	3–5 days
Signs and Symptoms	Fever, chills, headache, malaise, myalgias **Ulceroglandular**: lymphadenopathy, ulceration **Oculoglandular**: photophobia, conjunctivitis, lymphadenopathy **Oropharyngeal**: pharyngitis, exudate, cervical adenopathy **Pneumonic**: cough, chest pain, hilar adenopathy **Typhoidal**: combination of symptoms
Lab Findings	Non-specific and may or may not be present: leukocytosis, elevated inflammatory markers, thrombocytopenia, hyponatremia, transaminitis, elevated CPK (Creatine Phosphokinase), myoglobinuria, pyuria
Diagnosis	Isolation from specimen (PCR, positive antibody titer)
Treatment/Prophylaxis	**Adults**: Streptomycin, doxycycline **Children**: streptomycin **Alternative for adults and children**: gentamicin, ciprofloxacin

**Table t5-jetem-8-2-sg1:**

Disease: Ehrlichiosis (*E. chaffeensis*, *E. ewingii*, *E. muris eauclairensis*)	
Vector	*A. americanum* (lone star tick)
Endemic Area	Southeastern and South-central US
Incubation Period	5–14 days
Signs and Symptoms	Fever, chills, headache, malaise, myalgia, GI symptoms, altered mental status, rash
Lab Findings	Thrombocytopenia Leukopenia Anemia Transaminitis
Diagnosis	DNA PCR of whole blood Antibody titers Staining of sample tissue
Treatment/Prophylaxis	Doxycycline (5–7 days or for at least 3 days after resolution of fever)

**Table t6-jetem-8-2-sg1:**

Disease: Anaplasmosis	
Vector	*Ixodes scapularis*
Endemic Area	Woodlands, Eastern US
Incubation Period	1–2 weeks post exposure
Signs and Symptoms	Febrile illness without rash
Lab Findings	Leukopenia Lymphopenia Thrombocytopenia Mild transaminitis
Diagnosis	Nucleic acid amplification testing
Treatment/Prophylaxis	Doxycycline

**Table t7-jetem-8-2-sg1:**

Disease: Tick Paralysis	
Vector	*Dermacentor variabilis*
Endemic Area	Broadly distributed along East and West coasts of US
Incubation Period	4–7 days after attachment
Signs and Symptoms	Neurologic deficits Ascending paralysis
Lab Findings	No lab diagnosis
Diagnosis	Physical exam finding of tick
Treatment/Prophylaxis	Removal of tick

**Table t8-jetem-8-2-sg1:**

Disease: Rocky Mountain Spotted Fever	
Vector	*Dermacentor variabilis*
Endemic Area	Broadly distributed on the east and west coasts of the US
Incubation Period	2–14 days post exposure
Signs and Symptoms	High fever Flu-like illness with rash, but rash may be absent 10% of cases GI symptoms.
Lab Findings	No helpful lab findings
Diagnosis	Indirect immunofluorescence antibody
Treatment/Prophylaxis	Doxycycline

**Table t9-jetem-8-2-sg1:**

Disease: Powassan Virus	
Vector	*Ixodes scapularis*
Endemic Area	Northeast and Great Lakes regions
Incubation Period	1 week to 1 month
Signs and Symptoms	Many asymptomatic Fever Headache Vomiting Weakness Confusion Ataxia
Lab Findings	No helpful lab findings
Diagnosis	Serum or cerebrospinal fluid ELISA testing for Powassan IgM
Treatment/Prophylaxis	Supportive care

[Table t2-jetem-8-2-sg1] [Table t3-jetem-8-2-sg1] [Table t4-jetem-8-2-sg1] [Table t5-jetem-8-2-sg1] [Table t6-jetem-8-2-sg1] [Table t7-jetem-8-2-sg1] [Table t8-jetem-8-2-sg1] [Table t9-jetem-8-2-sg1]

## References

[1] Werner SL Banda BK Burnsides CL Stuber AJ Zoonosis: Update on existing and emerging vector-borne illnesses in the USA Curr Emerg Hosp Med Rep 2019 7 3 91 106 32288973 10.1007/s40138-019-00189-y PMC7102350

[2] Fuchs S Tick-Borne Infections Pediatric emergency care 2021 37 11 570 575 34731875 10.1097/PEC.0000000000002558

[3] Ho BM Davis HE Forrester JD Wilderness Medical Society Clinical Practice Guidelines for the Prevention and Management of Tick-Borne Illness in the United States Wilderness & Environmental Medicine 2021 32 4 474 494 34642107 10.1016/j.wem.2021.09.001

[4] Carrero E Gomar C Penzo W Rull M Comparison between lecture-based approach and case/problem-based learning discussion for teaching pre-anaesthetic assessment Eur J Anaesthesiol Dec 2007 24 12 1008 15 10.1017/S0265021506002304 17261212

[5] Thomas PA Kern DE Hughes MT Chen BY Curriculum development for medical education: a six-step approach JHU Press; 2016 10.1097/ACM.0000000000002580 30681454

[6] Naparin H Saad AB Infographics in education: Review on infographics design The International Journal of Multimedia & Its Applications (IJMA) 2017 9 4 5

[7] Merriam SB Bierema LL Adult learning: Linking theory and practice John Wiley & Sons 2013

[8] Reio TG Rocco TS Smith DH Chang E A critique of Kirkpatrick’s evaluation model New Horizons in Adult Education and Human Resource Development 2017 29 2 35 53

[9] Tickborne Diseases of the United States: A Reference Manual for Health Care Providers 2022 Centers for Disease Control and Prevention Available at: https://www.cdc.gov/ticks/tickbornediseases/TickborneDiseases-P.pdf

[10] Biggs HM Behravesh CB Bradley KK Diagnosis and Management of Tickborne Rickettsial Diseases: Rocky Mountain Spotted Fever and Other Spotted Fever Group Rickettsioses, Ehrlichioses, and Anaplasmosis — United States MMWR Recomm Rep 2016 65 RR-2 1 44 doi: 10.15585/mmwr.rr6502a1 27172113

[11] Wormser GP Dattwyler RJ Shapiro ED Lyme disease, human granulocytic anaplasmosis, and babesiosis: clinical practice guidelines by the Infectious Diseases Society of America. The clinical assessment, treatment, and prevention of Lyme disease, human granulocytic anaplasmosis, and babesiosis: clinical practice guidelines by the Infectious Diseases Society of America Clin Infect Dis 2006 43 1089 1134 17029130 10.1086/508667

[12] Swaminathan A Beck-Esmay J CORE EM Episode 44- Tick Borne Illnesses https://coreem.net/podcast/episode-44-0/

[13] Todd SR Dahlgren FS Traeger MS No visible dental staining in children treated with doxycycline for suspected Rocky Mountain spotted fever J Pediatr 2015 166 5 1246 1251 25794784 10.1016/j.jpeds.2015.02.015

[14] Eisen R Paddock C Ebel GD Pritt BS Emerging Tickborne Diseases. (Video.) YouTube July 2019 Centers for Disease Control and Prevention Available at: https://www.cdc.gov/grand-rounds/pp/2017/20170321-tickborne-diseases.html

[15] Cabrera D Tick season is now upon us! EMBlog Mayo Clinic June 2015 Available at: http://emblog.mayo.edu/2015/06/15/tick-season-is-now-upon-us/

